# Stable Co-Occurrence and Time-Specific Within-Person Associations Among Depressive Symptoms, Fear of Negative Evaluation, and Problematic Internet Use in Chinese Adolescents

**DOI:** 10.3390/bs16091689

**Published:** 2026-09-19

**Authors:** Zhenhua Li, Zijian Zheng, Yangang Nie, Pei Chen

**Affiliations:** Research Center of Adolescent Psychology and Behavior, School of Education, Guangzhou University, 230 Outer Ring West Road, Guangzhou Higher Education Mega Center, Guangzhou 510006, China; lzhua@gzhu.edu.cn (Z.L.); 13532603580@163.com (Z.Z.)

**Keywords:** problematic internet use, fear of negative evaluation, depressive symptoms, adolescents, random-intercept cross-lagged panel model

## Abstract

With the internet becoming increasingly integrated into adolescents’ lives, problematic internet use (PIU) is closely associated with their psychological adjustment. However, the longitudinal relationships between PIU, depressive symptoms (DS), and fear of negative evaluation (FNE) remain unclear. Therefore, based on three-wave longitudinal data from a convenience sample of 1062 Chinese adolescents (among those with available age data, ages ranged from 11 to 16 years; *M* = 13.71, *SD* = 1.58) in three secondary schools in Guangzhou, China, this study employed a random-intercept cross-lagged panel model to examine the longitudinal associations between PIU, DS, and FNE, and further tested gender differences in the structural pathways. Valid data on at least one core study variable were available for 1060, 881, and 883 participants at T1, T2, and T3, respectively. PIU, DS, and FNE were significantly positively associated at the between-person level, suggesting a stable co-occurrence of these three constructs among Chinese adolescents. However, they did not form stable, reciprocal within-person longitudinal associations across the two measurement intervals; instead, the significant associations were specific to particular intervals. Specifically, higher-than-usual PIU at T1 was associated with higher subsequent DS at T2, and higher-than-usual DS at T2 was associated with higher subsequent PIU and FNE at T3; in contrast, FNE was not significantly associated with subsequent PIU in either of the two adjacent time periods. Exploratory multigroup analysis did not provide evidence of an overall gender difference in structural paths. These findings preliminarily suggest that understanding adolescent PIU may benefit from distinguishing between stable between-person patterns across the observed study period and time-specific within-person changes.

## 1. Introduction

Problematic internet use (PIU) refers to a condition in which maladaptive online behaviors lead to negative consequences for individuals in psychological, social, and academic domains (Caplan, 2002; Sánchez-Fernández et al., 2023). A recent meta-analysis of Chinese adolescents revealed an overall prevalence of 10.3% (Zheng et al., 2025), indicating that adolescent PIU has become an emerging public health issue. Therefore, clarifying the psychological factors involved in adolescent PIU may provide useful evidence for its early identification and prevention.

However, adolescent PIU is not merely a behavioral issue related to internet use; it often interacts with an individual’s emotions, social relationships, and cognitive processes. PIU is associated with various psychosocial factors, including depressive symptoms, anxiety, social connectedness, self-esteem, negative emotion regulation, and fear of negative evaluation (FNE) (Cai et al., 2023; Ding et al., 2023; Naidu et al., 2023; Wang, 2022; Xu et al., 2024). Among these factors, depressive symptoms (DS) and FNE warrant particular attention. The former reflects adolescents’ emotional distress, whereas the latter reflects social-evaluative concerns. Previous studies have reported significant associations between PIU and depressive symptoms in adolescents, while FNE has also been linked to problematic internet or social networking use in young people (Cai et al., 2023; Naidu et al., 2023; Soriano-Molina et al., 2025; Xu et al., 2024). It remains uncertain whether these observed associations primarily arise from stable between-person differences or from fluctuations occurring within the same adolescent over time.

In other words, stable between-person differences cannot be directly inferred as within-person changes. Adolescents with consistently high levels of DS or FNE may also have higher levels of PIU, but this does not imply that within-person changes in DS or FNE will necessarily predict subsequent changes in PIU. Existing cross-sectional studies and traditional longitudinal models often struggle to distinguish between these two processes. Associations observed across individuals do not necessarily indicate that comparable changes occur within an individual over time (Maxwell & Cole, 2007). By separating stable interindividual differences from intraindividual fluctuations, the random-intercept cross-lagged panel model (RI-CLPM) provides a more appropriate framework for examining how the variables relate to one another over time.

Accordingly, the present study applies an RI-CLPM to PIU, DS, and FNE. Rather than treating their longitudinal associations as a single overall relationship, we separately examine stable differences between adolescents and time-specific fluctuations within adolescents.

### 1.1. PIU and DS

DS refer to a set of emotional symptoms characterized by low mood, reduced interest or pleasure, and related difficulties in sleep, appetite, and attention (Birmaher & Brent, 2007; Thapar et al., 2012). A systematic review indicates that DS not only hinder adolescents’ adaptive development but also adversely affect subsequent academic performance (Wickersham et al., 2021). Cross-sectional studies and meta-analyses generally indicate that adolescent DS are significantly positively associated with PIU (Chi et al., 2019; Xu et al., 2024; Ye et al., 2023), and longitudinal studies also suggest that DS often predict subsequent PIU (Leo et al., 2021; Villanueva-Blasco et al., 2026).

From the perspective of the vulnerability–stress model, higher levels of DS may indicate that adolescents are more vulnerable in terms of emotion regulation and stress coping (Ingram & Luxton, 2005; Monroe & Simons, 1991). Under real-life stress, these adolescents with elevated DS may turn to the internet to regulate negative affect or obtain temporary relief from stress, which may further increase the risk of subsequent PIU (Young & de Abreu, 2010).

Simultaneously, PIU may also contribute to subsequent DS. The time-displacement hypothesis posits that adolescents’ excessive internet use may displace time allocated to healthy activities such as sleep, academic engagement, and offline socializing (Röhlke, 2025). Existing research also indicates that PIU is associated with sleep disturbances, poorer mental health, and declining academic performance (Chen et al., 2025; Gül et al., 2022; Mei et al., 2018; Soriano-Molina et al., 2025). These adverse consequences may further undermine adolescents’ real-life adjustment and increase the risk of subsequent DS.

### 1.2. PIU and FNE

FNE refers to an individual’s anxiety about negative evaluations in social situations, the resulting distress, and a tendency to avoid such situations (Watson & Friend, 1969/1970). As a key cognitive feature of social anxiety, FNE is closely associated with the processing of negative social information, avoidant coping, and social insecurity (Fredrick & Luebbe, 2024; Leary, 1983; Morrison & Heimberg, 2013). Peer status and social evaluation become increasingly important during adolescence, resulting in a potentially greater focus on peer acceptance, feedback, and evaluation (Brown & Larson, 2009; Mitic et al., 2021). Therefore, FNE may serve as a significant source of social-evaluative concerns influencing adolescents’ online and offline social adaptation.

According to Kardefelt-Winther’s (2014) compensatory internet use theory, individuals may seek compensatory gratification through the internet when facing stress or negative affect in real life. For adolescents with a high FNE, face-to-face interactions may impose greater evaluative pressure than those occurring online. Compared with offline interactions, online environments typically offer greater control and may reduce the sense of threat posed by immediate social evaluation. Consequently, these adolescents may be more inclined to use online interactions to avoid real-life stress and obtain alternative social gratification, thereby increasing the risk of PIU (Kardefelt-Winther, 2014; Lee & Stapinski, 2012; Naidu et al., 2023). Existing research also indicates an association between FNE and problematic internet or social networking use (Peng et al., 2020; Zsido et al., 2020).

However, most existing studies examine the relationship between FNE and PIU or problematic social media use from a unidirectional predictive perspective, focusing on how FNE increases an individual’s risk of PIU (Casale et al., 2024; Hutcheson et al., 2025). In contrast, direct evidence regarding whether PIU, in turn, reinforces subsequent FNE remains limited. Some longitudinal studies have provided indirect clues from the perspective of social anxiety; for example, Hernández et al. (2024) found that PIU can predict subsequent social anxiety symptoms, and FNE is one of the key cognitive components of social anxiety. At the same time, PIU may further intensify adolescents’ concerns about negative evaluations by increasing their exposure to online feedback, social comparison, and others’ judgments (Nesi et al., 2018a, 2018b). However, the association between FNE and PIU does not necessarily manifest as a direct within-person longitudinal relationship. Therefore, it is necessary to distinguish between stable between-person co-occurrence and within-person longitudinal associations between the two using RI-CLPM.

### 1.3. Integrative Relationships Between PIU, DS, and FNE

Previous studies have considered the relationships between DS and PIU and between FNE and PIU; however, these two sets of relationships may not be independent of one another. Theoretically, they may influence PIU through psychological vulnerability and maladaptive cognitive processes, respectively. Davis’s (2001) cognitive–behavioral model of pathological internet use posits that PIU is closely related to an individual’s psychological state and cognitive processing styles, rather than being determined solely by internet use behavior itself. Based on this perspective, DS and FNE may be related to PIU through two distinct mechanisms: DS reflect adolescents’ emotional distress, while FNE reflects their anxiety regarding negative evaluations from others. Both may be associated with higher levels of PIU; however, whether they contribute to the within-person longitudinal associations with PIU in the same way requires further examination.

Specifically, DS and FNE may be correlated with each other and jointly reflect the internalizing risk in adolescents. Therefore, when examining the pairwise relationships (DS–PIU and FNE–PIU), it is difficult to determine whether the association of DS and FNE with PIU reflects their independent effects or primarily reflects common variations among the three variables at the level of stable between-person differences. By simultaneously modeling PIU, DS, and FNE, the RI-CLPM enables us to separately examine stable between-person differences across the observed study period and fluctuations within adolescents over time.

### 1.4. Current Study

**Figure 1:**
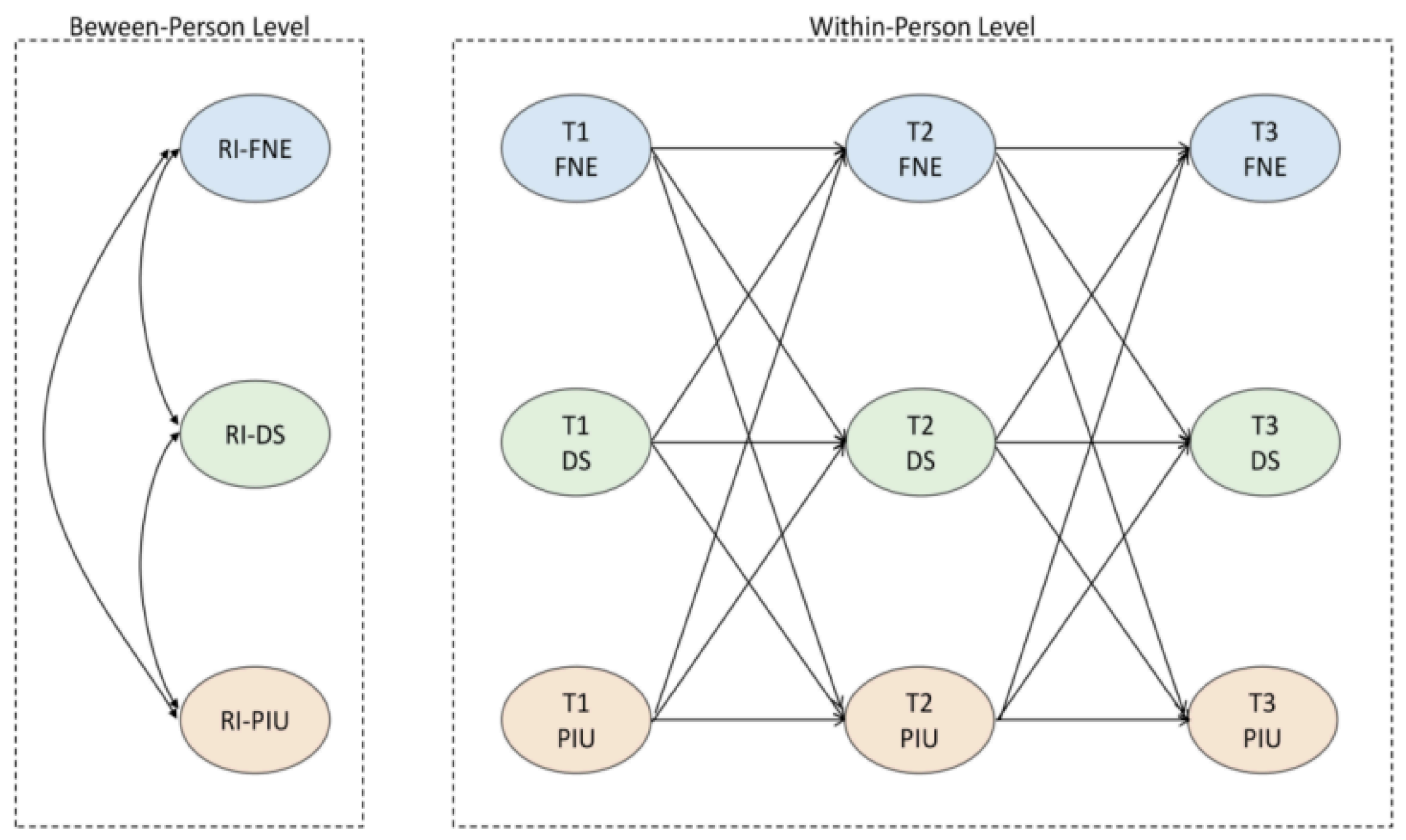
Random Intercept Cross-Lagged Panel Model for Depressive Symptoms (DS), Problematic Internet Use (PIU), and Fear of Negative Evaluation (FNE) Across Three Waves. Note. RI = random intercept; T1 = Wave 1 (November 2021); T2 = Wave 2 (May 2022); T3 = Wave 3 (May 2023). Single-headed arrows represent autoregressive and cross-lagged paths, whereas double-headed curved arrows represent correlations among the random intercepts.

Based on three-wave longitudinal data, we employ the RI-CLPM (Figure 1) to examine the longitudinal associations between PIU, DS, and FNE among Chinese adolescents. Specifically, we examine whether stable co-occurrence exists among these three variables at the between-person level and further investigate whether longitudinal associations emerge at the within-person level across the two measurement intervals. In addition, exploratory multigroup analyses are conducted to examine whether structural paths differ by gender.

Based on prior theory and empirical evidence, we propose the following hypotheses:

**H1a.**  *At the within-person level, DS positively predicts subsequent PIU.*

**H1b.**  *At the within-person level, higher-than-usual PIU positively predicts subsequent higher-than-usual DS.*

**H2a.**  *At the within-person level, higher-than-usual FNE positively predicts subsequent higher-than-usual PIU.*

**H2b.**  *At the within-person level, higher-than-usual PIU positively predicts subsequent higher-than-usual FNE.*

**H3.**  *At the between-person level, the random intercepts of PIU, DS, and FNE are positively correlated, indicating stable co-occurrence among the three constructs.*

## 2. Materials and Methods

### 2.1. Participants and Procedure

This study was a three-wave longitudinal observational study. The three secondary schools in Guangzhou, China, were selected through convenience sampling. The final analytic sample comprised 302 (28.4%), 352 (33.1%), and 408 (38.4%) students from the three participating schools, respectively. Participants were students in Grade 7 (*n* = 524, 49.3%) and Grade 10 (*n* = 538, 50.7%). Among participants with available age data, ages ranged from 11 to 16 years, with a mean age of 13.71 years (*SD* = 1.58). All students in the selected classes were invited to participate. Students were eligible to participate if they were enrolled in the selected classes and parental or legal-guardian consent and student assent were obtained. No additional exclusion criteria were applied at the recruitment stage. No a priori sample-size calculation or power analysis was conducted. The sample size was determined by the number of students in the selected classes who participated in the school-based survey. A total of 1374 students from the selected classes participated in the study. Of these, 1062 provided valid data on at least one core study variable at one or more measurement waves and were included in the final analytic sample. The remaining 312 participants did not provide a valid score on any of the three core study variables (PIU, DS, or FNE) at any of the three measurement waves and were therefore not included in the analytic sample. Because the longitudinal models were estimated using full information maximum likelihood (FIML), participants with partially observed longitudinal data were retained rather than excluded solely because of missing data at a particular wave. At T1, T2, and T3, respectively, 1060 (99.8%), 881 (83.0%), and 883 (83.1%) participants provided valid data on at least one core study variable. Among the 1060 participants with valid T1 data, 779 (73.5%) provided valid data at all three waves, whereas 281 (26.5%) had incomplete follow-up data at T2 and/or T3. Two additional participants had no valid core-variable data at T1 but provided valid data at both T2 and T3. Having a valid core-variable score at T1 was not required for inclusion in the final analytic sample; participants with valid core-variable data at later waves were retained and contributed their available data to the longitudinal analyses. Participant flow and wave-specific data availability are summarized in Supplementary Figure S1.

Students participated in the survey in November 2021 (T1), May 2022 (T2), and May 2023 (T3). The originally planned December 2022 measurement could not be conducted owing to COVID-19-related campus management arrangements; consequently, the T1–T2 interval was approximately 6 months, whereas the T2–T3 interval was approximately 12 months. Before data collection, the research team explained the study’s objectives, confidentiality procedures, and matters related to voluntary participation to parents or legal guardians. Written informed consent was obtained from the parents or legal guardians, and written assent was obtained from the students. All assessments were conducted on-site at the school, with participants completing the questionnaires in person as a class. Two trained graduate students were assigned to each classroom to assist with the administration, explain the questionnaire completion requirements, answer procedural questions, and ensure the standardization of the administration process. This project was approved by the institutional review board of the authors’ affiliated institution.

### 2.2. Measures

All instruments were administered in Chinese.

#### 2.2.1. FNE

FNE was measured using the Brief FNE Scale (BFNE) developed by Leary (1983). The original 12-item version comprises eight positively worded items and four negatively worded items. Given that the negatively worded items in the BFNE may form a method factor and affect the stability of the scale’s structure (Carleton et al., 2006; Rodebaugh et al., 2004/2005), later studies supported the BFNE-Straightforward version, which contains only eight positively worded items (Carleton et al., 2011; Wei et al., 2015). Therefore, following the BFNE-Straightforward approach, this study used only the eight straightforwardly worded items to assess adolescents’ FNE. Responses are rated from 1 (“not at all”) to 5 (“very much”), yielding total scores between 8 and 40, with higher scores reflecting greater FNE. The Cronbach’s α coefficients for the scale at T1, T2, and T3 were 0.922, 0.925, and 0.935, respectively.

#### 2.2.2. DS

DS were assessed with the 10-item Center for Epidemiologic Studies Depression Scale developed by Andresen et al. (1994). The Andresen 10-item short form has also demonstrated satisfactory factor structure and criterion validity among Chinese adolescents (Yang et al., 2018). The scale consists of 10 items, including eight items assessing DS and two positive affect items that are reverse coded. Each item is rated on a 4-point scale from 1 (“rarely or none of the time”) to 4 (“most or all of the time”). Total scores range from 10 to 40, with higher values indicating greater depressive symptom severity. The Cronbach’s α coefficients at T1, T2, and T3 were 0.850, 0.863, and 0.852, respectively.

#### 2.2.3. PIU

PIU was measured using Li et al.’s (2010) 10-item Chinese scale, which is based on Young’s (1998) revised diagnostic criteria for internet addiction. This scale is used to assess adolescents’ maladaptive internet use behaviors and core PIU symptoms, including excessive preoccupation with internet use, tolerance, loss of control, withdrawal-like reactions, spending more time online than intended, functional impairment, concealing the extent of Internet use, and using the internet to alleviate negative affect. Responses are rated on a 6-point scale from 1 (“completely disagree”) to 6 (“completely agree”). The item scores are combined to yield a total ranging from 10 to 60, with higher values reflecting greater PIU symptom severity. This scale measures general PIU and does not further distinguish specific types of online activities such as online gaming, online shopping, social networking, short video use, or general browsing. The Cronbach’s α coefficients at T1, T2, and T3 were 0.89, 0.91, and 0.91, respectively. It should be noted that the PIU scores in this study reflect a continuous tendency toward PIU symptoms, rather than an internet use disorder in the context of a clinical diagnosis. For all three measures, scale scores were calculated only when all constituent items were available. If one or more items were missing, the corresponding scale score at that wave was treated as missing. No item-level imputation or prorating was performed.

### 2.3. Data Analysis

Data were analyzed in the following steps. First, SPSS 26.0 was used to summarize PIU, DS, FNE, age, and gender and to estimate the bivariate correlations among these variables.

Second, attrition was examined prior to the main longitudinal analysis among the 1060 participants who provided valid data on at least one core study variable at T1. Participants who provided valid data on at least one core study variable at all three waves (*n* = 779) were compared with those who had valid T1 data but incomplete follow-up data at T2 and/or T3 (*n* = 281). Group differences in age and baseline PIU, DS, and FNE were evaluated using independent-samples t-tests, whereas gender distributions were compared using a chi-square test. Because some baseline variables contained variable-specific missing data, the available sample size differed across individual attrition comparisons. Missing scale scores in the RI-CLPM were handled using full information maximum likelihood (FIML) under the missing-at-random assumption. Thus, participants with partially observed longitudinal data contributed all available scale-level information to model estimation, whereas missing individual items were not imputed.

Third, longitudinal measurement invariance tests, the candidate RI-CLPMs, and multigroup RI-CLPMs were conducted in Mplus 8.3. Longitudinal measurement invariance was examined for PIU, DS, and FNE across the three waves at the configural, metric, strong, and strict levels. Residuals of the same items across measurement waves were allowed to correlate in the longitudinal measurement invariance models. Model fit was evaluated based on the comparative fit index (CFI), Tucker–Lewis index (TLI), root mean square error of approximation (RMSEA), and standardized root mean square residual (SRMR), and changes in fit between adjacent invariance models were assessed using ΔCFI and ΔRMSEA.

After confirming the comparability of the scales across time, this study established an RI-CLPM based on the total scale scores at each time point to examine the temporal associations among PIU, DS, and FNE. Thus, longitudinal measurement invariance was first examined at the item level, whereas the subsequent RI-CLPM was estimated using observed total scale scores rather than item-level latent measurement models. In the primary full-sample model, age and gender were included as covariates and specified as predictors of the observed scores at each wave, allowing their associations with the repeated measures to vary across measurement occasions rather than constraining their effects to operate solely through stable between-person components. Gender was coded as 1 = boy, 2 = girl. All longitudinal models were estimated using maximum likelihood with robust standard errors (MLR). Because time-invariant covariates can be incorporated into RI-CLPMs in different ways (Mulder & Hamaker, 2021) and covariate placement may affect the decomposition of between-person and within-person variance, an alternative specification in which age and gender predicted the random intercepts was additionally examined as a sensitivity analysis.

To determine appropriate model specifications, this study established four RI-CLPMs based on the degree of path constraints: M1 set the autoregressive paths, cross-lagged paths, and within-wave residual correlations to be equal across time; M2 allowed the cross-lagged paths to be freely estimated while constraining the autoregressive paths and within-wave residual correlations to be equal; M3 further allowed the autoregressive paths to be freely estimated, while the within-wave residual correlations remained equal across time; M4, building on M3, further relaxed selected equality constraints on the within-wave covariance structure. Specifically, the T1 within-person covariances among DS, PIU, and FNE were freely estimated rather than constrained equal to the corresponding follow-up covariances. In addition, the concurrent residual covariance between DS and PIU was freely estimated at T2 and T3, whereas the corresponding DS–FNE and PIU–FNE residual covariances remained constrained equal across the two follow-up waves. Given that the interval between T1–T2 was approximately 6 months and that between T2–T3 was approximately 12 months, equality constraints on autoregressive paths across the two intervals were considered difficult to justify substantively. Therefore, M3 was selected as the primary model, whereas M2 was retained as a more parsimonious constrained comparison model. Model selection considered global model fit indices, Akaike information criterion (AIC), Bayesian information criterion (BIC), model parsimony, and the substantive plausibility of the imposed temporal constraints.

After determining the primary model, multigroup RI-CLPMs were further used to test for gender differences in structural paths. Specifically, models with freely estimated structural paths were first estimated for the male and female groups. Subsequently, the corresponding structural paths for the two groups were set to be equal, and the fit differences between the free and constrained models were compared using a robust chi-square difference test based on MLR.

## 3. Results

### 3.1. Missing Data and Attrition

Partial item-level missingness was low across the three measures and measurement waves, ranging from 0.7% to 1.6% of the analytic sample. Because scale scores were calculated only when all constituent items were available, any partial item-level missingness resulted in a missing scale score for that wave. Among the 1060 participants who provided valid data on at least one core study variable at T1, 779 provided valid data at all three waves, whereas 281 had incomplete follow-up data at T2 and/or T3. Supplementary Table S1 summarizes the attrition analysis. Compared with adolescents with valid data at all three waves, those with incomplete follow-up data were significantly older, t(545.86) = −10.49, *p* < 0.001, d = 0.70; had higher baseline PIU scores, t(1047) = −3.09, *p* = 0.002, d = 0.22; and had slightly higher baseline DS scores, t(895) = −1.98, *p* = 0.048, d = 0.15. The groups did not differ significantly in baseline FNE, t(887) = −1.19, *p* = 0.233, d = 0.09. Gender distribution also differed significantly, χ^2^(1, *N* = 1049) = 28.35, *p* < 0.001, φ = 0.16, with boys more frequently represented among participants with incomplete follow-up. Because baseline age, gender, PIU, DS, and FNE contained different amounts of missing data, the available sample size varied across these comparisons. These findings indicate some selective attrition, particularly with respect to age and gender. FIML was used to retain all available longitudinal information under the missing-at-random assumption.

### 3.2. Descriptive Statistics and Correlation Analyses

Among participants with available age data, ages ranged from 11 to 16 years (*M* = 13.71, *SD* = 1.58). Among those with available gender data, 575 (54.7%) were boys and 476 (45.3%) were girls. The descriptive statistics for each variable are presented in Table 1. The mean values of PIU, DS, and FNE across the three waves of data showed a slight upward trend over time. Furthermore, the three core variables were largely significantly positively associated both within the same measurement wave and across different measurement waves, indicating a consistent pattern of bivariate associations.

### 3.3. Longitudinal Measurement Invariance

Table 2 presents the results of the longitudinal measurement invariance tests for PIU, DS, and FNE. For FNE, invariance tests were based on the retained eight straightforwardly worded items. Across configural, metric, strong, and strict invariance models, all three constructs showed acceptable model fit (CFI ≥ 0.901, RMSEA ≤ 0.048, and SRMR ≤ 0.056; TLI values ranged from 0.892 to 0.936). Comparisons between adjacent models also showed small changes in model fit (ΔCFI ≤ 0.01, ΔRMSEA ≤ 0.015). Overall, the results provided acceptable support for strict longitudinal measurement invariance across the three waves, although model fit for DS was somewhat weaker, with TLI values slightly below 0.90.

### 3.4. RI-CLPM Results

The results of the model comparison are shown in Table 3, and the specific constraint settings for the four candidate RI-CLPMs are presented in Supplementary Table S2. All four candidate models showed good global fit. Given the unequal measurement intervals between T1–T2 (approximately 6 months) and T2–T3 (approximately 12 months), M3 was selected as the primary model because it allowed the autoregressive paths to vary freely across the two intervals rather than imposing equality constraints across substantially different lag lengths. M3 showed good global model fit, with χ^2^(15) = 34.964, RMSEA = 0.035, CFI = 0.993, TLI = 0.973, and SRMR = 0.034. Although M2 was more parsimonious and yielded slightly lower AIC and BIC values, its equality constraints on autoregressive paths were considered less substantively appropriate given the unequal measurement intervals. Therefore, M2 was retained as a constrained comparison model rather than the primary model.

**Figure 2:**
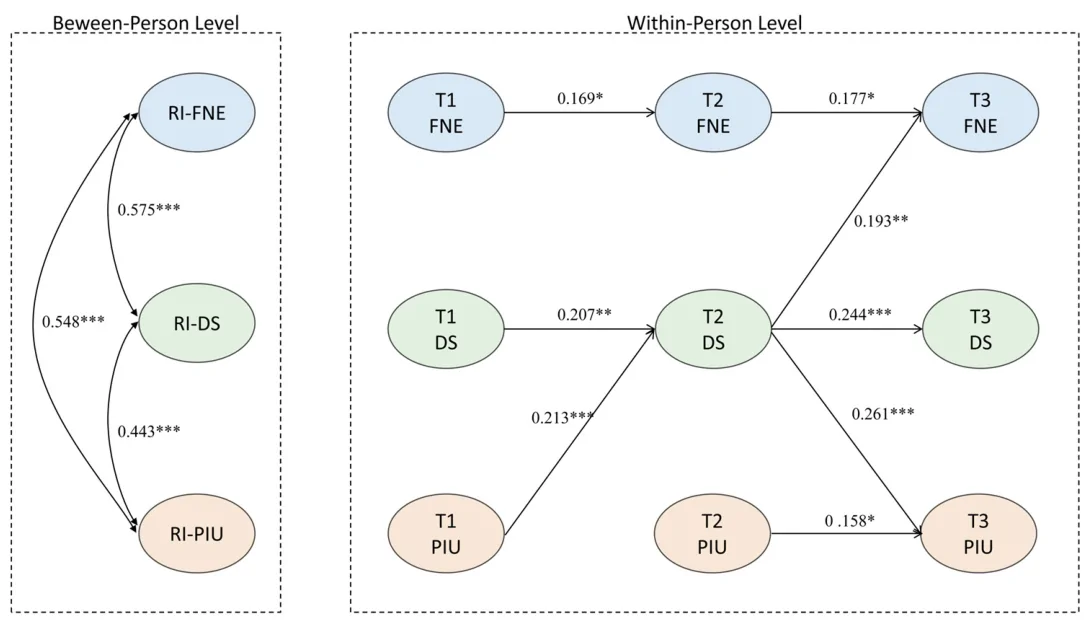
Final RI-CLPM Results for Depressive Symptoms (DS), Problematic Internet Use (PIU), and Fear of Negative Evaluation (FNE) Across Three Waves. Note. RI = random intercept; T1 = Wave 1 (November 2021); T2 = Wave 2 (May 2022); T3 = Wave 3 (May 2023). Estimates are from the primary M3 RI-CLPM (*N* = 1062). Single-headed arrows represent significant autoregressive or cross-lagged paths, whereas double-headed curved arrows represent significant correlations among the random intercepts. Only significant paths are displayed. Values are standardized coefficients (STDYX). * *p* < 0.05, ** *p* < 0.01, *** *p* < 0.001. Age and gender were included as covariates but are omitted from the figure for clarity.

M4 further relaxed selected equality constraints on the within-wave covariance structure compared with M3. Specifically, the T1 within-person covariances among DS, PIU, and FNE were freely estimated rather than constrained equal to the corresponding follow-up covariances, and the concurrent DS–PIU residual covariance was estimated separately at T2 and T3, whereas the DS–FNE and PIU–FNE residual covariances remained constrained equal across the two follow-up waves. Although M4 showed relatively favorable values for several fit indices, it introduced additional free parameters and had fewer degrees of freedom. Given the three-wave design and the absence of a strong substantive rationale for these additional relaxations, M4 was not preferred over M3. Accordingly, M3 was retained as the primary model, with M2 serving as the constrained comparison model. Specific path estimates from M3 are shown in Figure 2 and Table 4.

At the between-person level, the random intercepts of DS, PIU, and FNE were all significantly positively associated, indicating stable between-person co-occurrence among the three variables. Specifically, RI-DS was significantly positively associated with RI-PIU (β = 0.443, *p* < 0.001) and RI-FNE (β = 0.575, *p* < 0.001), and RI-PIU was also significantly positively associated with RI-FNE (β = 0.548, *p* < 0.001).

At the within-person level, the autoregressive effects varied across constructs and intervals. DS showed significant autoregressive effects from T1 to T2 (β = 0.207, *p* = 0.005) and from T2 to T3 (β = 0.244, *p* < 0.001). FNE also showed significant autoregressive effects across both intervals (T1 → T2: β = 0.169, *p* = 0.047; T2→T3: β = 0.177, *p* = 0.047). For PIU, the autoregressive effect from T1 to T2 was not significant (β = 0.129, *p* = 0.093), whereas the effect from T2 to T3 was significant (β = 0.158, *p* = 0.022). Regarding the cross-lagged effects, PIU at T1 was significantly positively associated with DS at T2 (β = 0.213, *p* < 0.001). DS at T2, in turn, was significantly positively associated with PIU at T3 (β = 0.261, *p* < 0.001) and FNE at T3 (β = 0.193, *p* = 0.003). The remaining cross-lagged paths were not statistically significant. The standardized coefficients of the three significant cross-lagged associations ranged from 0.193 to 0.261, indicating modest effect magnitudes overall, with the DS T2 → PIU T3 association showing the largest standardized coefficient (β = 0.261). In particular, FNE was not significantly associated with subsequent PIU across either interval. Overall, these findings indicate that the within-person longitudinal associations among PIU, DS, and FNE were time-specific rather than consistently reciprocal across the two intervals.

As a sensitivity analysis, age and gender were alternatively specified as predictors of the random intercepts rather than the wave-specific observed scores. This alternative specification showed poorer global fit, χ^2^(27) = 246.146, CFI = 0.919, TLI = 0.838, RMSEA = 0.087, and SRMR = 0.082. Nevertheless, the three focal cross-lagged associations remained significant: PIU T1 → DS T2 (β = 0.225, *p* < 0.001), DS T2 → PIU T3 (β = 0.281, *p* < 0.001), and DS T2 → FNE T3 (β = 0.203, *p* = 0.002), while FNE remained unrelated to subsequent PIU across both intervals. However, PIU T2 → DS T3 also became significant (β = 0.167, *p* = 0.003), indicating that some within-person estimates were sensitive to covariate placement.

Analysis of covariates revealed that age was significantly positively associated with PIU across all three waves (T1: β = 0.178, *p* < 0.001; T2: β = 0.175, *p* < 0.001; T3: β = 0.170, *p* < 0.001) and with FNE across all three waves (T1: β = 0.143, *p* < 0.001; T2: β = 0.130, *p* < 0.001; T3: β = 0.134, *p* < 0.001) and showed significant positive associations with DS at T2 and T3 (T2: β = 0.062, *p* = 0.026; T3: β = 0.067, *p* = 0.016), but the association with DS at T1 (β = 0.044, *p* = 0.133) was nonsignificant. Gender was significantly positively associated with DS across all three waves (T1: β = 0.166, *p* < 0.001; T2: β = 0.180, *p* < 0.001; T3: β = 0.218, *p* < 0.001) and with FNE across all three waves (T1: β = 0.176, *p* < 0.001; T2: β = 0.235, *p* < 0.001; T3: β = 0.233, *p* < 0.001) and showed significant positive associations with PIU at T2 and T3 (T2: β = 0.073, *p* = 0.015; T3: β = 0.129, *p* < 0.001), but the association with PIU at T1 (β = 0.014, *p* = 0.636) was nonsignificant. As the coding scheme was boys = 1 and girls = 2, these results collectively indicate that girls scored significantly higher than boys on DS and FNE at all three time points, as well as on PIU at T2 and T3.

To further explore potential gender differences in the longitudinal structural paths, multigroup RI-CLPMs were estimated based on the primary M3 specification. The multigroup analysis included 575 boys and 476 girls with available gender data (*N* = 1051). The freely estimated model showed good fit, χ^2^(30) = 53.854, RMSEA = 0.039, SRMR = 0.046, CFI = 0.990, and TLI = 0.971. A constrained model in which the corresponding 18 autoregressive and cross-lagged paths were set equal across boys and girls also showed good fit, χ^2^(48) = 71.657, RMSEA = 0.031, SRMR = 0.046, CFI = 0.990, and TLI = 0.982. The MLR-scaled chi-square difference test was nonsignificant, Δχ^2^(18) = 17.72, *p* = 0.474, indicating that imposing equality constraints on the longitudinal structural paths did not significantly worsen model fit. Thus, under the present sample and model specification, this exploratory analysis did not provide evidence of an overall gender difference in the longitudinal structural paths among DS, PIU, and FNE. These findings should nevertheless be interpreted cautiously, as the multigroup analysis was exploratory and a nonsignificant overall difference test should not be taken as evidence that the structural relations are identical across gender.

## 4. Discussion

Using an RI-CLPM, this study examined the longitudinal associations among DS, PIU, and FNE while separating stable between-person differences from within-person fluctuations. At the between-person level, the random intercepts of PIU, DS, and FNE were all significantly positively associated, supporting H3 and indicating stable co-occurrence among the three constructs. At the within-person level, PIU at T1 was significantly associated with subsequent DS at T2, whereas DS at T2 was significantly associated with subsequent PIU at T3. However, the corresponding reverse-direction paths within the same intervals were not significant, indicating that H1a and H1b received only partial and time-specific support rather than evidence of a stable reciprocal relationship. In contrast to H2a and H2b, FNE was not significantly associated with subsequent PIU across either interval, nor was PIU significantly associated with subsequent FNE. DS at T2 was additionally associated with subsequent FNE at T3. Exploratory multigroup analysis did not provide evidence of an overall gender difference in the longitudinal structural paths. Overall, these findings highlight the importance of distinguishing stable between-person co-occurrence from time-specific within-person longitudinal associations when interpreting the psychological correlates of adolescent PIU.

### 4.1. Stable Between-Person Co-Occurrence Among PIU, DS, and FNE

At the between-person level, the random intercepts of DS, PIU, and FNE were significantly positively associated, supporting H3. This suggests that adolescents who consistently exhibit high levels on one construct also tend to exhibit consistently high levels on other constructs; for example, adolescents with higher DS scores often report higher levels of PIU and FNE. In other words, PIU, DS, and FNE showed a clear pattern of stable co-occurrence at the between-person level across the observed study period. This suggests that the risks associated with adolescent PIU are not solely manifested in online behavior itself but are also linked to psychological factors such as emotional distress and social-evaluative concerns. However, stable between-person associations cannot be directly interpreted as within-person longitudinal associations. In other words, adolescents with consistently high levels of DS also tend to report higher levels of PIU, but this does not necessarily mean that a within-person increase in DS will be followed by a subsequent increase in PIU. For this reason, the key value of RI-CLPMs lies in distinguishing stable between-person differences from within-person fluctuations over time, thereby avoiding the misinterpretation of stable between-person differences as within-person changes (Hamaker, 2024; Hamaker et al., 2015; Mulder & Hamaker, 2021).

### 4.2. Time-Specific Within-Person Associations Between PIU and DS

The results partially supported H1a and H1b, indicating time-specific within-person longitudinal associations between DS and PIU. Specifically, PIU at T1 was significantly associated with DS at T2, whereas DS at T2 was significantly associated with PIU at T3. However, the corresponding reverse-direction paths were not significant within the same intervals. Previous longitudinal studies have suggested that the association between PIU and DS may vary over time (Kojima et al., 2021; Villanueva-Blasco et al., 2026; Yi & Li, 2021). The present findings further suggest that these within-person associations were not consistently reciprocal across the two observed intervals, but instead differed across measurement periods.

The significant association between PIU at T1 and DS at T2 suggests that when adolescents’ PIU is higher than their usual level at a given measurement occasion, their subsequent DS is also more likely to be higher than their usual level. One possible explanation is that PIU may not merely be a manifestation of adolescents’ emotional distress but may also be associated with subsequent emotional adjustment problems. Some longitudinal studies have also found that PIU can predict subsequent DS (Kojima et al., 2021; Villanueva-Blasco et al., 2026). Furthermore, existing research suggests that PIU may be associated with real-life adjustment problems such as sleep issues, impaired academic functioning, and reduced offline interaction, factors that may in turn increase the risk of DS (Gül et al., 2022; Ma & Sheng, 2023; Mei et al., 2018). However, this study did not directly examine these specific mechanisms; therefore, the underlying mechanisms linking PIU to subsequent DS require further investigation in future research.

The significant association between DS at T2 and PIU at T3 is largely consistent with compensatory internet use theory. Specifically, within-person increases in DS may reflect stronger negative affect and greater adjustment pressure at a given measurement occasion. Under such conditions, adolescents may be more inclined to adopt avoidant or compensatory coping strategies, using the internet as a tool to escape real-life stress, alleviate negative affect, or obtain alternative social gratification, thereby increasing the risk of subsequent PIU (Davis, 2001; Kardefelt-Winther, 2014). It is worth noting that even after simultaneously controlling for FNE, the association between DS at T2 and PIU at T3 remained significant. This result suggests that, within the present sample, higher-than-usual DS at T2 was associated with higher subsequent PIU at T3.

Finally, the pattern of cross-lagged associations between PIU and DS was not consistently reciprocal across the two intervals, suggesting that their relationship should not be interpreted as a stable or continuous bidirectional process. One possible reason is that longitudinal associations may depend on the length of the measurement interval and the broader context in which the assessments occur. In the present study, the interval between T1 and T2 was approximately 6 months, whereas that between T2 and T3 was approximately 12 months. These unequal lag lengths may have influenced the magnitude and statistical significance of the estimated paths, making direct comparisons across intervals difficult. Therefore, the present findings are more appropriately interpreted as time-specific within-person longitudinal associations between PIU and DS rather than evidence of a stable bidirectional cycle. It should also be noted that PIU T2 → DS T3 became significant under the alternative covariate specification, suggesting that part of the within-person pattern was sensitive to covariate placement.

### 4.3. FNE and PIU: Stable Between-Person Co-Occurrence Without Direct Within-Person Cross-Lagged Associations

In contrast to H2a and H2b, this study did not provide evidence for direct within-person cross-lagged associations between FNE and PIU. Specifically, FNE was not significantly associated with subsequent PIU across either interval, and PIU likewise was not significantly associated with subsequent FNE. At the same time, the random intercepts of FNE and PIU were significantly positively associated, indicating stable between-person co-occurrence despite the absence of significant direct within-person cross-lagged associations during the observed periods.

One possible interpretation is that adolescents with higher FNE across the observed study period may also tend to report higher PIU because both are related to broader patterns of social-evaluative concern, avoidance, or maladaptive coping (Lee & Stapinski, 2012; Naidu et al., 2023). However, the present RI-CLPM cannot determine the mechanisms underlying this stable between-person association. At the within-person level, within-person elevations in FNE may also operate through more proximal processes, such as social anxiety, avoidance, online feedback seeking, self-control difficulties, or emotion-regulation difficulties (Peng et al., 2020; Zsido et al., 2020), none of which were directly measured in the present study. Therefore, the current findings should not be interpreted as showing that FNE is unimportant for PIU; rather, they indicate that FNE did not show a direct within-person cross-lagged association with subsequent PIU across the two observed intervals.

Notably, DS at T2 was significantly associated with FNE at T3. This result suggests that, at the within-person level, subsequent changes in FNE may be related to DS. Existing research indicates that DS are typically accompanied by negative self-evaluation, social withdrawal, and poor emotional adjustment, and these characteristics may increase adolescents’ concerns about others’ evaluations (Birmaher & Brent, 2007; Fredrick & Luebbe, 2024; Morrison & Heimberg, 2013; Thapar et al., 2012). In this study, DS at T2 was associated with both subsequent PIU and subsequent FNE at T3. This pattern suggests that within-person elevations in DS may be relevant to understanding subsequent changes in both PIU and FNE, although the mechanisms underlying these associations remain to be examined.

It should be emphasized that this study did not formally test the mediation effects between PIU, DS, and FNE; therefore, this pattern cannot be interpreted as a strict mediational chain. A more prudent interpretation is that, in the present three-wave RI-CLPM, FNE and PIU were stably associated at the between-person level, but FNE did not show a direct within-person cross-lagged association with subsequent PIU; in contrast, DS was more closely associated with changes in both subsequent PIU and FNE.

### 4.4. Gender Differences in the Longitudinal Associations Between PIU, DS, and FNE

Exploratory multigroup RI-CLPM analysis did not provide evidence of an overall gender difference in the longitudinal structural paths among PIU, DS, and FNE. Specifically, constraining the corresponding autoregressive and cross-lagged paths to equality across boys and girls did not significantly worsen model fit. However, this nonsignificant overall difference test should not be interpreted as evidence that the structural relations are identical across gender. Given the exploratory nature of the multigroup analysis, future studies with larger samples and more measurement waves are needed to further examine whether specific longitudinal pathways vary by gender.

### 4.5. Practical Implications

This study offers several practical implications for understanding and identifying psychological risks associated with adolescent PIU. First, given the time-specific within-person associations observed between PIU and DS, assessments of PIU risk may benefit from considering not only internet use duration or online behaviors but also changes in depressive symptoms and broader emotional adjustment. Importantly, the present findings do not indicate a stable bidirectional relationship between PIU and DS; rather, the direction of their longitudinal associations differed across the two observed intervals.

Second, within-person elevations in DS at T2 were associated with subsequent increases in both PIU and FNE at T3. This pattern suggests that changes in depressive symptoms may represent a potentially relevant emotional indicator when assessing adolescents’ subsequent internet-use difficulties and social-evaluative concerns. These findings may inform future research examining whether prevention and support efforts related to PIU could benefit from considering adolescents’ emotional distress, stress coping, and emotion-regulation difficulties in addition to their online behavior. However, because the present study was observational, these findings should not be interpreted as evidence that interventions targeting DS would necessarily reduce subsequent PIU or FNE.

Finally, although FNE was not significantly associated with subsequent PIU, FNE and PIU were stably associated at the between-person level. This suggests that FNE may remain a relevant contextual characteristic for understanding the broader psychological profile associated with adolescent PIU, even though it did not show a direct within-person cross-lagged association with subsequent PIU in the present study. Therefore, assessments of adolescents with persistently elevated PIU may also consider social-evaluative concerns such as sensitivity to peer feedback and concerns about others’ evaluations.

### 4.6. Limitations and Future Directions

This study has several limitations. First, although this study used three-wave longitudinal data and an RI-CLPM to separate stable between-person differences from within-person fluctuations, the observed longitudinal associations should not be interpreted as causal effects. In addition, the measurement intervals were unequal, with approximately 6 months between T1 and T2 and 12 months between T2 and T3. Although the primary model allowed the autoregressive paths to vary freely across the two intervals rather than imposing equality constraints, the unequal lag lengths may still have influenced the magnitude and statistical significance of the longitudinal associations and therefore limit direct comparisons across intervals. Moreover, the sensitivity analysis examining alternative covariate placement showed that the three focal cross-lagged associations remained significant, but an additional PIU T2 → DS T3 path also became significant, indicating that some within-person estimates were sensitive to model specification. Data collection was also affected by the COVID-19 pandemic, and adolescents’ internet use and psychological states may have been influenced by this broader context. Future studies should use more evenly spaced measurement occasions, additional waves, and alternative model specifications to further examine the robustness of these longitudinal associations.

Second, selective attrition should be considered when interpreting the findings. Among the 1060 participants who provided valid data on at least one core study variable at T1, 779 provided valid data at all three waves, whereas 281 had incomplete follow-up data at T2 and/or T3. Compared with participants with valid data at all three waves, those with incomplete follow-up were older, included a higher proportion of boys, and showed slightly higher baseline PIU and DS scores. Although FIML was used to incorporate all available longitudinal data and age and gender were included as covariates, the missing-at-random assumption cannot be directly verified, and selective attrition related to the study variables themselves may still have biased the longitudinal estimates. Future studies should implement measures to improve participant retention and further examine the robustness of these longitudinal associations across different age and gender groups.

Third, the sample was drawn through convenience sampling from three secondary schools in Guangzhou, China; therefore, caution is warranted when generalizing the results to Chinese adolescents in other regions, school types, or cultural contexts. Additionally, this study included two cohort groups—7th and 10th graders—and students at different educational levels may face varying developmental tasks, academic pressures, and school contexts. Although age was controlled for in the model, cohort- or grade-level heterogeneity was not directly modeled, and it remains necessary to further examine whether these longitudinal associations vary across grade levels or educational stages.

Fourth, all variables were based on student self-reports, which may introduce some degree of self-report and common-method bias. Future studies could incorporate multi-source information, such as screen-use data, digital behavior trajectories, and parent or teacher reports, to enhance the objectivity of the measurements.

Finally, this study examined general PIU and did not further distinguish between specific types of online activities, such as social media, online gaming, short videos, and general web browsing. Consequently, we could not determine whether different digital activities share similar longitudinal pathways with DS and FNE. Future research could further distinguish between different types of online activities and incorporate variables such as social anxiety (Ding et al., 2023), social connectedness (Cai et al., 2023), and self-esteem (Wang, 2022) to construct a more comprehensive developmental mechanism framework.

## 5. Conclusions

Using three-wave longitudinal data, this study employed an RI-CLPM to examine the associations among PIU, DS, and FNE at both the between-person and within-person levels. The three constructs showed stable positive co-occurrence at the between-person level, whereas their within-person longitudinal associations were time-specific rather than consistently reciprocal across the two observed intervals. Specifically, higher-than-usual PIU at T1 was associated with higher subsequent DS at T2, whereas higher-than-usual DS at T2 was associated with higher subsequent PIU and FNE at T3. In contrast, FNE did not show a direct within-person cross-lagged association with subsequent PIU across either interval. These findings underscore the importance of distinguishing stable between-person differences across the observed study period from within-person fluctuations when interpreting psychological correlates of adolescent PIU. They also suggest that changes in depressive symptoms may be particularly relevant for understanding subsequent changes in PIU and FNE, while the association between FNE and PIU may be more evident as stable between-person co-occurrence than as a direct within-person longitudinal association. Given the unequal lengths of the two follow-up intervals and the selective attrition observed across waves, these interval-specific findings should be interpreted cautiously and replicated in future longitudinal studies.

## Figures and Tables

**Table 1:** Means, Standard Deviations, and Correlations of Study Variables.

Variable	1	2	3	4	5	6	7	8	9	10	11
1. Age	1										
2. Gender	−0.12 **	1									
3. T1 PIU	0.20 **	0.00	1								
4. T2 PIU	0.17 **	0.06	0.57 **	1							
5. T3 PIU	0.12 **	0.09 **	0.53 **	0.59 **	1						
6. T1 DS	−0.01	0.16 **	0.42 **	0.27 **	0.28 **	1					
7. T2 DS	0.06	0.20 **	0.39 **	0.35 **	0.39 **	0.66 **	1				
8. T3 DS	0.00	0.21 **	0.23 **	0.28 **	0.42 **	0.51 **	0.61 **	1			
9. T1 FNE	0.16 **	0.17 **	0.46 **	0.32 **	0.33 **	0.48 **	0.42 **	0.32 **	1		
10. T2 FNE	0.08 *	0.25 **	0.36 **	0.38 **	0.37 **	0.42 **	0.50 **	0.36 **	0.62 **	1	
11. T3 FNE	0.06	0.22 **	0.26 **	0.26 **	0.42 **	0.32 **	0.41 **	0.46 **	0.52 **	0.59 **	1
*M*	13.71	1.45	27.27	29.74	30.99	18.63	20.03	20.78	24.82	25.36	25.84
*SD*	1.58	0.50	10.61	10.91	10.77	5.89	6.24	5.93	7.84	7.80	7.83
*N*	1017	1051	1049	869	875	897	874	866	889	786	865

Note. *M* = mean; *SD* = standard deviation; *N* = number of participants with valid data for each variable. Pearson correlation coefficients are reported. PIU = problematic Internet use. DS = depressive symptoms. FNE = fear of negative evaluation. T1 = Wave 1 (November 2021); T2 = Wave 2 (May 2022); T3 = Wave 3 (May 2023). Gender was coded as 1 = boy and 2 = girl. Sample sizes varied across variables because of missing data. * *p* < 0.05, ** *p* < 0.01.

**Table 2:** Longitudinal Measurement Invariance of PIU, DS, and FNE Across Three Waves.

Variable	Model	CFI	ΔCFI	RMSEA	ΔRMSEA	TLI	SRMR
PIU	Configural invariance	0.931	—	0.046	—	0.919	0.035
	Metric invariance	0.930	−0.001	0.046	0.000	0.922	0.038
	Strong invariance	0.925	−0.005	0.046	0.000	0.920	0.039
	Strict invariance	0.921	−0.004	0.046	0.000	0.920	0.041
DS	Configural invariance	0.908	—	0.048	—	0.892	0.052
	Metric invariance	0.907	−0.001	0.047	−0.001	0.897	0.053
	Strong invariance	0.902	−0.005	0.047	0.000	0.896	0.053
	Strict invariance	0.901	−0.001	0.046	−0.001	0.899	0.056
FNE	Configural invariance	0.944	—	0.048	—	0.932	0.034
	Metric invariance	0.943	−0.001	0.047	−0.001	0.935	0.036
	Strong invariance	0.939	−0.004	0.048	0.001	0.934	0.036
	Strict invariance	0.937	−0.002	0.047	−0.001	0.936	0.041

Note. PIU = problematic Internet use; DS = depressive symptoms; FNE = fear of negative evaluation. For FNE, longitudinal measurement invariance was examined based on the retained eight straightforwardly worded items. CFI = comparative fit index; TLI = Tucker–Lewis index; RMSEA = root mean square error of approximation; SRMR = standardized root mean square residual.

**Table 3:** Model Fit Indices for the Candidate RI-CLPMs.

Model	χ^2^	df	CFI	TLI	RMSEA	SRMR	AIC	BIC
M1	53.078	24	0.989	0.976	0.034	0.041	57,847.141	58,110.440
M2 ^b^	34.383	18	0.994	0.982	0.029	0.034	57,838.023	58,131.130
M3 ^a^	34.964	15	0.993	0.973	0.035	0.034	57,844.001	58,152.011
M4	21.876	11	0.996	0.980	0.031	0.017	57,835.059	58,162.941

Note. χ^2^ = chi-square statistic; df = degrees of freedom; CFI = comparative fit index; TLI = Tucker–Lewis index; RMSEA = root mean square error of approximation; SRMR = standardized root mean square residual; AIC = Akaike information criterion; BIC = Bayesian information criterion. ^a^ Primary model with freely estimated autoregressive paths due to unequal intervals between waves. ^b^ Constrained comparison model with autoregressive paths constrained to be equal across intervals.

**Table 4:** RI-CLPM Estimates of the Longitudinal Associations Among Depressive Symptoms, Problematic Internet Use, and Fear of Negative Evaluation.

Section	Path	β	95% CI	*p*
Between-person correlations	**RI-DS ↔ RI-PIU**	**0.443**	**[0.345, 0.541]**	**<0.001**
	**RI-DS ↔ RI-FNE**	**0.575**	**[0.465, 0.685]**	**<0.001**
	**RI-PIU ↔ RI-FNE**	**0.548**	**[0.434, 0.662]**	**<0.001**
Stability effects	**DS T1 → DS T2**	**0.207**	**[0.066, 0.349]**	**0.005**
	**DS T2 → DS T3**	**0.244**	**[0.110, 0.378]**	**<0.001**
	PIU T1 → PIU T2	0.129	[−0.022, 0.280]	0.093
	**PIU T2 → PIU T3**	**0.158**	**[0.025, 0.292]**	**0.022**
	**FNE T1 → FNE T2**	**0.169**	**[0.004, 0.334]**	**0.047**
	**FNE T2 → FNE T3**	**0.177**	**[0.000, 0.354]**	**0.047**
Cross-lagged effects	**PIU T1 → DS T2**	**0.213**	**[0.105, 0.320]**	**<0.001**
	FNE T1 → DS T2	0.094	[−0.033, 0.221]	0.145
	DS T1 → PIU T2	0.052	[−0.079, 0.182]	0.436
	FNE T1 → PIU T2	−0.024	[−0.158, 0.109]	0.721
	DS T1 → FNE T2	0.127	[−0.019, 0.274]	0.087
	PIU T1 → FNE T2	0.076	[−0.051, 0.203]	0.241
	PIU T2 → DS T3	0.078	[−0.042, 0.198]	0.204
	FNE T2 → DS T3	−0.013	[−0.150, 0.123]	0.849
	**DS T2 → PIU T3**	**0.261**	**[0.146, 0.377]**	**<0.001**
	FNE T2 → PIU T3	0.042	[−0.100, 0.184]	0.566
	**DS T2 → FNE T3**	**0.193**	**[0.067, 0.319]**	**0.003**
	PIU T2 → FNE T3	−0.031	[−0.151, 0.089]	0.611

Note. β values and 95% confidence intervals are STDYX-standardized estimates. *p* values are based on the corresponding unstandardized parameter tests. DS = depressive symptoms; PIU = problematic Internet use; FNE = fear of negative evaluation; RI = random intercept. T1 = Wave 1 (November 2021); T2 = Wave 2 (May 2022); T3 = Wave 3 (May 2023). → indicates a directional autoregressive or cross-lagged path, whereas ↔ indicates a between-person random-intercept correlation. Estimates are from the primary M3 RI-CLPM (*N* = 1062). Significant effects (*p* < 0.05) are shown in bold. Confidence interval bounds are rounded to three decimal places.

## Data Availability

The datasets generated during and/or analyzed during the current study are available from the corresponding author on reasonable request.

## Supplementary Materials

The following supporting information can be downloaded at https://www.mdpi.com/article/10.3390/bs16091689/s1, Figure S1: Participant flow and wave-specific data availability across the three-wave study; Table S1: Attrition analyses comparing adolescents with valid data at all three waves with those with incomplete follow-up data; Table S2: Specifications of the four candidate RI-CLPMs.

## Abbreviations

The following abbreviations are used in this manuscript:

PIU	Problematic internet use
DS	Depressive symptoms
FNE	Fear of negative evaluation
BFNE	Brief Fear of Negative Evaluation Scale
CFI	Comparative fit index
RMSEA	Root mean square error of approximation
TLI	Tucker–Lewis index
SRMR	Standardized root mean square residual
RI-CLPM	Random-intercept cross-lagged panel model
AIC	Akaike information criterion
BIC	Bayesian information criterion
MLR	Maximum Likelihood with Robust standard errors
FIML	Full information maximum likelihood
