# Supplementary material for: Stable Co-Occurrence and Time-Specific Within-Person Associations Among Depressive Symptoms, Fear of Negative Evaluation, and Problematic Internet Use in Chinese Adolescents
Source: Behav Sci (Basel). 2026 Sep 19;16(9):1689. doi: 10.3390/bs16091689 (PMC13603843; doi:10.3390/bs16091689)
Supplement: Supplementary file 1 [file behavsci-16-01689-s001.zip › behavsci-4535232-supplementary.pdf]

Supplementary Materials

Table S1. Attrition analyses comparing adolescents with valid data at all three waves with those with incomplete follow-up data.

Baseline variable	Valid data at all three waves	Incomplete follow-up	Test	*p*	Effect size
Age	13.43 ± 1.56	14.48 ± 1.36	$t(545.86) = -10.49$	< .001	$d = 0.70$
PIU T1	26.66 ± 10.49	28.95 ± 10.80	$t(1047) = -3.09$	.002	$d = 0.22$
DS T1	18.38 ± 5.80	19.25 ± 6.09	$t(895) = -1.98$	.048	$d = 0.15$
FNE T1	24.63 ± 7.95	25.32 ± 7.54	$t(887) = -1.19$	.233	$d = 0.09$
Gender, boys %	49.8%	68.3%	$\chi^2(1, N = 1049) = 28.35$	< .001	$\varphi = 0.16$

Note. PIU = problematic internet use; DS = depressive symptoms; FNE = fear of negative evaluation. For age, Welch's t test is reported because Levene's test indicated unequal variances. For PIU, DS, and FNE, equal-variance t tests are reported. Gender was coded as 1 = boy and 2 = girl.

Table S2. Specifications of the four candidate RI-CLPMs.

Model	Baseline orthogonality constraints	Autoregressive paths	Cross-lagged paths	Within-wave residual correlations
M1	Imposed	Constrained equal	Constrained equal	Constrained equal
M2[b]	Imposed	Constrained equal	Freely estimated	Constrained equal
M3[a]	Imposed	Freely estimated	Freely estimated	Constrained equal
M4	Imposed	Freely estimated	Freely estimated	Partially freed

Note. “Constrained equal” indicates that corresponding parameters were constrained to be equal across adjacent intervals. Baseline orthogonality constraints refer to constraints involving the random intercepts and the Wave 1 within-person latent factors. In M4, the baseline orthogonality constraints remained imposed. The T1 within-person covariances among DS, PIU, and FNE were freely estimated rather than constrained equal to the corresponding follow-up covariances. The concurrent DS–PIU residual covariance was freely estimated separately at T2 and T3, whereas the DS–FNE and PIU–FNE residual covariances remained constrained equal across the two follow-up waves. [a] Primary model with freely estimated autoregressive paths due to unequal intervals between waves. [b] Constrained comparison model with autoregressive paths constrained to be equal across intervals.

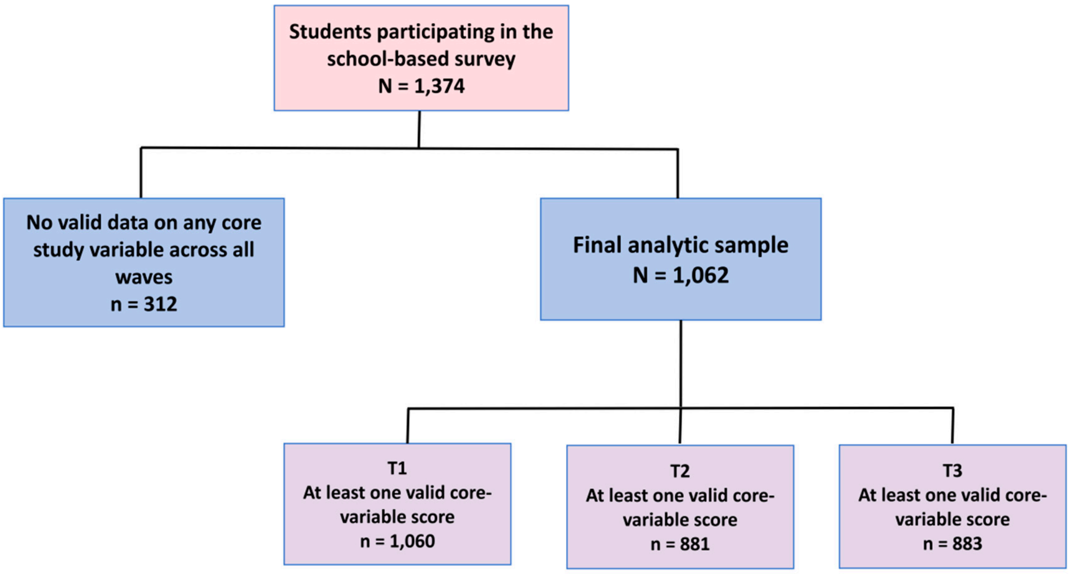

Figure S1. Participant Flow and Wave-Specific Data Availability Across the Three-Wave Study. Note. Valid data at each wave were defined as having a valid score on at least one of the three core study variables: problematic internet use (PIU), depressive symptoms (DS), or fear of negative evaluation (FNE). T1 = November 2021; T2 = May 2022; T3 = May 2023. Two participants in the final analytic sample had no valid core-variable data at T1 but provided valid data at both T2 and T3; therefore, the T1 sample size was 1,060 rather than 1,062.
